# Programmed cell death-1 and its ligands: Current knowledge and possibilities in immunotherapy

**DOI:** 10.1016/j.clinsp.2023.100177

**Published:** 2023-03-15

**Authors:** Bojana Gutic, Tatjana Bozanovic, Aljosa Mandic, Stefan Dugalic, Jovana Todorovic, Dejana Stanisavljevic, Miroslava Gojnic Dugalic, Demet Sengul, Dzenana A. Detanac, Ilker Sengul, Dzemail Detanac, José Maria Soares

**Affiliations:** a Oncology Institute of Vojvodina, Faculty of Medicine University Novi Sad, Faculty of Medicine, University of Belgrade, Belgrade, Serbia; b Department of Gynecology and Obstetrics, University Clinical Center of Serbia, Clinic for Gynecology and Obstetrics, Belgrade, Serbia; c Institute for Social Medicine, Faculty of Medicine, University of Belgrade, Belgrade, Serbia; d Institute for Statistics, Faculty of Medicine, University of Belgrade, Belgrade, Serbia; e Department of Pathology, Faculty of Medicine, Giresun University, Giresun, Turkey; f Department of Ophthalmology, General Hospital Novi Pazar, Novi Pazar, Serbia; g Division of Endocrine Surgery, Faculty of Medicine, Giresun University, Giresun, Turkey; h Department of Surgery, Faculty of Medicine, Giresun University, Giresun, Turkey; i Department of Surgery, General Hospital Novi Pazar, Novi Pazar, Serbia; j Faculdade de Medicina da Universidade de São Paulo, São Paulo, SP, Brazil

**Keywords:** Programmed cell death, Ligands, Immunohistochemistry, Immunology, Pathology

## Abstract

Programmed Cell Death-1 (PCD-1) is a key immune checkpoint receptor, which mainly expresses on activated T, B, Dendritic (DC), Natural Killer (NK), and Treg cells. On the surface of activated T-cells, PCD-1 expression is upregulated after the recognition of peripherals antigens by T cells; subsequently, the elevated binding of PD-1 to Programmed Death Ligand-1 (PD-L1) and Programmed Death Ligand-2 (PD-L2) becomes a key step for downstream inhibitory signaling. Although the role of PD-L1 has been evaluated more thoroughly by clinical research, and PD-L1 has also been used more widely in the clinical setting, PD-L2 also plays an important role in the negative regulation of T-cells, one of the necessary conditions that lead to immune tolerance. Expression of PD-L1 either in tumors or in infiltrating immune cells has been verified predominantly by Immunohistochemistry (IHC) in a variety of tumors, suggesting a role for the PD-1/PD-L1 axis as a prognostic trait and therapeutic target across multiple histotypes. The complex interplay between these factors plays a major role in the diffusion and clinical application of PD-L1 IHC assays as predictive biomarkers of response to PD-1/PD-L1 inhibitors. Checkpoint blockades are registered for the treatment of various cancers, including gynecological malignancies.

## Highlights

• Recently, cancer immunotherapy targeting PD-1 or PD-L1 has proven effective in causing durable antitumor immune responses with less toxicity in many types of tumors.
• PD-1/PD-L1 blockade therapy will be the major cancer immunotherapy modality.
• A deeper understanding of personal genomic information, and personalized markers in guiding anti-PD therapy.
• Similar to the tip of an iceberg, PD-1/PD-L1 blockade antitumor immunotherapy opens a new era of cancer treatment, and further work on safety and efficiency will be required.

## Introduction

A key immune checkpoint receptor, Programmed Cell Death-1 (PCD-1), principally expresses on some activated cells, called T, B, Dendritic (DC), Natural Killer (NK), and Treg. PCD-1 is also associated with increased Treg-cell proliferation and enhanced immunosuppressive function. A series of inflammatory cytokines, such as Interferon-gamma (IFN-γ), secreted by activated T-cells and NK cells, can promote PD-L1 expression in a variety of cell types, including epithelial, endothelial, hematopoietic, and tumor cells. Of note, oncogenes may also play an important role in stimulating the expression of PD-L1. In contrast, PD-L2 is expressed mainly in DCs, macrophages, bone marrow-derived mast cells, peritoneal B1 cells, helper T-cells, and intrapulmonary nonhematological cells. Similar to PD-L1, PD-L2 expression can be observed in some types of tumor cells. PD-L1 and PD-L2 expression are correlated in tumors; however, in some types of tumor cells, PD-L1 can be present in the absence of PD-L2, and vice versa.^2^^,^^3^ After the recognition of tumor antigens by peripheral T-Cell Receptors (TCRs), PD-L1 or PD-L2 will bind PD-1 and activate downstream-related signaling pathways, blocking TCR signaling via feedback inhibition, and downregulate the expression of specific antiapoptotic protein molecules including Bcl-xL, which is encoded by BCL2L1, that is one of the most common amplified genes among cancers, and pro-inflammatory factors, ultimately inhibiting T-cell survival, proliferation, and immune function. In addition, the interaction of PCD-1 with its ligand can also affect the cell cycle (by increasing the expression of p15 decreasing the transcription level of S-phase Kinase-associated Protein-2 (SKP2), precluding T-cells from entering the G1 phase, and eventually inhibiting cell proliferation), stimulating tumor-specific T-cell apoptosis and promote differentiation of T-helper (Th) cells into Tregs.^4^ The present investigation was approved by the Ethical Committee, University of Belgrade, Belgrade, Serbia, with 4/20/2-3707/2-6.

## Programmed cell death-1 and its ligands

Although the role of PD-L1 has been evaluated more thoroughly by clinical research, and PD-L1 has also been used more widely in the clinical setting, PD-L2 also plays an important role in the negative regulation of T cells, one of the necessary conditions that lead to immune tolerance.^5^ PD-L2 can also bind to the repulsive molecule b. Blockade of the repulsive molecule b-PD-L2 interaction impaired the development of respiratory tolerance by interfering with the initial T-cell expansion required for respiratory tolerance.^6^ Previous studies revealed that T-cell activity in PD-L2 knockout mice was enhanced, the amounts of the activated cluster of differentiation (CD)4+/CD8+ T-cells stimulated by exogenous antigen were augmented, and PD-L2 knockout also resulted in the disappearance of T-cell tolerance against foreign antigens.^5^ In general, the function of PD-L1 and PD-L2 overlap, but they also possess their unique features. PD-L1 has a priority in controlling the interaction of T-cells and antigen-presenting cells, inhibiting PD-L1-Enhanced hapten-induced Contact Hypersensitivity (CH) in a mouse model, and increasing the number of T-cells in draining lymph nodes, whereas PD-L2 inhibition did not show a similar strengthening effect.^7^ On the other hand, PD-L2 processes the ability to drive Th1 cell response through IFN-γ, and signaling of the PCD-1/PD-L2 pathway can significantly inhibit TCR-mediated CD4+ T-cell proliferation and cytokine production.^8^ Blocking PD-L2 on the surface of DC enhanced the proliferative capacity of T-cells and the production of cytokines such as IFN-γ and Interleukin-10 (IL-10).^4^ Dual inhibition of PD-L1 and PD-L2 can show a synergistic effect.^4^ Since the binding of PD-L1 and PD-L2 to PCD-1 is cross-competitive, when PD-1 inhibitors anchor the binding site and conformation of PCD-1, the interaction between PCD-1 and both PD-L1/PD-L2 ligands can be blocked simultaneously. Therefore, PCD-1 inhibitors are able to completely antagonize the PD-L1/PD-L2 axes, whereas PD-L1 inhibitors selectively block the PCD-1/PD-L1 mechanism, but do not impact PD-L2 binding to PCD-1. In addition to its interaction with PCD-1, PD-L1 can also bind to B7-1. Both B7-1 and B7-2 belong to the B7 family, which can interact with CD28 to initiate a series of stimulatory signals for T-cell activation. In the early stages of T-cell activation, Cytotoxic T Lymphocyte-Associated Antigen-4 (CTLA-4, another type of immune checkpoint molecule) can competitively bind with B7-1/B7-2 to inhibit CD28: B7 signaling, thereby preventing T-cell activation. B7-2 is constantly expressed at a low level on the surface of the antigen-presenting cell, and can be activated rapidly; in contrast, B7-1 only showed inducible expression after B7-2 activation. Previous studies have shown that PD-L1 can also bind competitively to B7-1, and there is an overlap between the binding interfaces of PD-L1: B7-1, CD28: B7-1, and CTLA-4: B7-1. Therefore, PD-L1 can inhibit T-cell activation and immune effector cytokine production, along with other inhibitory pathways such as CTLA-4. Although PD-L1 inhibitors disrupt the interaction between PD-L1 and B7-1, CTLA-4 may also compensatively disqualify this ‘immunosuppressive’ effect, leaving the immune system still at the inhibited status controlled by the checkpoint molecule. In addition, the interaction between CTLA-4: B7-1 and CD28: B7 signaling often plays an important role in the early stage of T-cell activation, which weakens, inhibits, and/or abrogates the activation of naive T-cells in the second lymphoid organs (such as lymph nodes), while the high expression of PD-L1 is often specifically limited to tumor cells and immune cells in the tumor microenvironment. Therefore, more evidence is still required to determine whether the inhibition of PD-L1 will affect the axis of PD-L1/B7-1/CD28 and the corresponding early activation process of T-cells.^9^

PD-L1 is encoded by the PDCDL1 gene and is found on chromosome 9 in humans at position p24.1.2 First described by Dong et al. in 1999 as B7-H1, PD-L1 was recognized as the third member of the B7 protein family, displaying a 15%–20% homology with B7.1 and B7.2 proteins.^3^ The full length of PD-L1 is encoded within seven exons, corresponding to a 40 kDa protein of 290 amino acids. PD-L1 is a type 1 transmembrane protein and consists of IgV-like and IgC-like extracellular domains, a hydrophobic transmembrane domain, and a short cytoplasmic tail made from 30 amino acids, with unclear signal transduction properties.^10^

PD-L1 expression can be constitutive or inducible. Constitutive, low-level PD-L1 expression can be found in resting lymphocytes, Antigen-Presenting Cells (APCs), and in corneal, syncytiotrophoblastic, and Langerhans’ islet cells where it contributes to tissue homeostasis in proinflammatory responses.^1^ PD-L1 confers certain tissues such as the placenta, testis, and the anterior chamber of the eye an ‘immune privileged’ status, where inoculation of exogenous antigens is tolerated without induction of an inflammatory/immune response. In the context of inflammation and/or infection, PD-L1 is induced as a suppressive signal on hematopoietic, endothelial, and epithelial cells.^6^ PD-L1 expression is primarily influenced by Toll-Like Receptors (TLRs), a subtype of non-catalytic receptors, highly expressed in APCs and activated by pathogen-associated molecular patterns. TLR-mediated regulation of PD-L1 relies on the activation of the MEK/ERK kinases, which enhance PD-L1 messenger RNA (mRNA) transcription via nuclear factor kappa B. IFN receptors 1 and 2 are also implicated in regulating PD-L1 expression, largely through Janus kinase (Jak)/Signal Transducer(s) and Activator(s) of Transcription (STAT)-mediated activation of Interferon Regulatory Factor-1 (IRF-1). Interferon-mediated activation of Jak/STAT can also up-regulate PD-L1 expression through the Mitogen-activated protein/Extracellular signal-Regulated Kinase (MEK/ERK) and the Phosphatidyl-Inositol-3 Kinase (PI3K)/ its downstream molecule serine/threonine Protein Kinase B (PKB; also known as AKT) pathway, which exerts a permissive role on PD-L1 transcription through phosphorylation of mammalian target of rapamycin.^11^

In carcinogenesis, PD-L1 can be overexpressed as a result of driver oncogenic events. Epidermal Growth Factor Receptor (EGFR) mutations, for instance, positively correlate with PD-L1 expression in lung cancer, with EGFR inhibitors acting as repressors of PD-L1 transcription.^8^ In Phosphatase and Tensin Homolog (PTEN)-mutant tumors, PD-L1 overexpression is sustained by unrestrained activation of the PI3K/AKT pathway.^9^ In T-cell lymphoma, the Nucleophosmin (NPM)/Anaplastic Lymphoma Kinase (ALK) fusion gene up-regulates PD-L1 via constitutive STAT3 activation.^12^

The PCD-1/PD-L1 pathway is crucial for the development of immune tolerance, a process of negative selection of autoreactive lymphocytes taking place in the primary (central tolerance) and secondary lymphoid organs (peripheral tolerance). High PD-L1 expression is, in fact, demonstrated within the thymus and on dendritic cells, where the PD-L1/PCD-1 interaction prevents the proliferation and differentiation of naïve T cells. Knock-out of PCD-1/PD-L1 leads to autoimmunity in animal models with lupus-like arthritis, glomerulonephritis, and diabetes. In humans, immune-related toxicity is a recognized class effect of anti-PCD-1/PD-L1 antibodies, where colitis, endocrinopathy, and immune/inflammatory dermatoses are common complications.^13^ Expression of PD-L1 either in tumors or in infiltrating immune cells has been verified predominantly by Immunohistochemistry (IHC) in a variety of tumors, suggesting a role for the PCD-1/PD-L1 axis as a prognostic trait and therapeutic target across multiple histotypes. However, IHC-based detection of PD-L1 expression is constrained by preanalytical and analytical variability including heterogeneity in antibody clones, scoring methodology, and intrinsic biological variation in PD-L1 expression due to the type of specimen analyzed (surgical resection vs. biopsy, primary tumor vs. metastasis, archival vs. fresh frozen) as well as prior treatment status. The complex interplay between these factors plays a major role in the diffusion and clinical application of PD-L1 Immunohistochemistry (IHC) assays as predictive biomarkers of response to PCD-1/PD-L1 inhibitors.^14^

While the clinical significance of PD-L1, the main ligand for PCD-1, has been widely investigated in cancer, the role of PD-L2 expressed by the immune, stromal, and tumor cells has received less attention and has been considered less relevant in predicting responses to immune checkpoint blockade with anti–PD-1/PD-L1 agents.^15^ The PCD-1/PD-Ls pathway plays a fundamental role in manipulating the magnitude of T-cell responses, regulating their activation, and generating immune tolerance in the Tumor Microenvironment (TME) and peripheral tissues. Furthermore, the PD-1 pathway controls humoral responses, where the activity of B-cells is modulated by follicular helper T-cells and follicular regulatory T-cells that were found positive for both PCD-1 and Cytotoxic T-Lymphocyte Antigen-4 (CTLA-4). Indeed, B-cells can express PD-L1 and PD-L2 but also CD80 and CD86, which are the respective ligands for PD-1 and CTLA-4 (CD152). Furthermore, PD-L2 expression was identified in a variety of tumor types even in the absence of PD-L1 expression,^16^ and a recent study revealed the presence of PD-L2 specific T-lymphocytes able to recognize their targets expressed by either tumor or immune cells and able to induce the release of Th-1 cytokines, like IFN-γ and Tumor Necrosis Factor-alpha (TNF-α). PD-L2 can be found on immune cells, including B lymphocytes (where it can bind to PD-1 on follicular helper T-cells, resulting in reduced long-lived plasma cell number, and on dendritic cells as well as on other types of hematopoietic and nonhematopoietic cells.^17^ Physiologically, PD-L2, together with PD-L1, plays a role in the regulation of mucosal CD4+ T-cell responses when expressed by colonic myofibroblasts, a cell population that is major histocompatibility complex class II positive (MHC-II+). These PD-L2+ cells exert suppressive functions concerning activated CD4+ Th cell proliferation by inhibiting Interleukin (IL)-2, whose production can be increased after PD-1/PD-L1 or PD-1/PD-L2 blockade. In addition, PD-L2 can be expressed on macrophages, bone marrow-derived mast cells, more than 50% of peritoneal B1 lymphocytes, and intestinal stromal cells (exerting a role in immune tolerance). PD-L2 expression on dendritic cells is induced by IL-4 and Granulocyte-Monocyte Colony-Stimulating Factor (GM-CSF).^18^ PD-L2 expression was found on activated CD1a+ dendritic cells in patients with cutaneous squamous cell carcinoma. It was usually associated with the presence of PD-L1 and with the size of the tumor. Further, PD-L2 was detected on either CD4+- or CD8+-specific T-lymphocytes that did not cross-react with PD-L1+–specific T lymphocytes in melanoma. These PD-L2–specific T-cells were able to react to autologous target cells depending on PD-L2 expression, directly supporting antitumor immunity by killing target cells and, indirectly, by releasing proinflammatory cytokines in the TME in response to PD-L2–expressing immunosuppressive cells. In clear cell renal cell carcinoma, high expression of PD-L2 together with PD-1, LAG3, and PD-L1 was associated with a more immunosuppressive TME.^19^ In breast cancer, membrane immunohistochemical expression of PD-L2 by immune cells, detected with the monoclonal antibody (clone = D7U8C) from Cell Signaling Technology, was found almost negative, with these findings being confirmed also by flow cytometry data here, the monoclonal antibody. In prostate cancer, deficient mismatch repair mutational signatures (found in 8.1% of the cohort) were associated with an increased extent of immune cells, immune checkpoint molecule detection, and T-lymphocyte-associated transcripts, including the expression of PD-L2 by antigen-presenting cells. In B-cell lymphomas, PD-L2 is usually detected on protumor M2 Tumor-Associated Macrophages (TAMs), and similar findings can be observed in classical Hodgkin lymphoma where high expression of PD-L2 (and PD-L1) by TAMs was associated with the suppression of the function of NK cells. In peripheral T-lymphocytes from non–small cell lung cancer patients, PD-L2 and PD-L1 expression were linked with higher levels of IL-2 and TNF-α and with worse Overall Survival (OS). In addition, tumor cells can express PD-L2. Preclinical models of renal cell carcinoma and lung squamous cell carcinoma reveal that tumor expression of PD-L2, through the inhibition of the activity of CD8+ T-cells, has a protumor role in the TME and could be involved in the resistance to anti–PCD-1 antibodies, which could be overcome by the combined use of anti–PCD-1 or anti–PD-L2 immune checkpoint blockade. Its significance was also confirmed in the clinical setting.^20^

A recent study identified a key Super-Enhancer (SE), PD-L1L2-SE, located between the encoding regions for PD-L1 and PD-L2. The activation of PD-L1L2-SE is required for PD-L1 and PD-L2 expression on tumor cells and genetic deletion of PD-L1L2-SE leads to tumor cell loss of immune evasion and increased sensitivity to T-cell killing. Interestingly, this mechanism of induction of PD-L1 and PD-L2 is independent of IFN-γ.^21^ In *Homo sapiens*, PD-L2 expression by tumor cells is probably associated with a Th2 response, mediated by IL-4 and IL-13, as shown in esophageal cancer and by IFN-γ in colorectal cancer. In melanoma cells, PD-L2 is responsive to IFN-beta (β) and IFN-γ and is regulated through the transcription factors IRF1 and STAT3 that bind to the PD-L2 promoter.^21^ PD-L2 presence is inversely associated with a Crohn-like lymphoid reaction in colorectal cancer probably inhibiting the development of tertiary lymphoid tissues. In small intestine and pancreatic neuroendocrine tumors, while the expression of PD-1 or PD-L1 was rare, that of PD-L2 was common. No clear associations between PD-L2, immune infiltrates, and specific mutational profiles within each tumor type were found. In gallbladder cancer, PD-L2 was more common than PD-L1 expression. In endometrial cancer, PD-L2 positivity was found on tumor cells in 64.4% and on immune cells in 93.2% of cases. In bladder cancer, 67% of the specimens exhibited PD-L2 positivity on tumor cells. PD-L2 expression by tumor cells was also observed in lung cancer, where it is usually associated with the detection of other immune checkpoint molecules (PD-L1, indoleamine-pyrrole 2,3-dioxygenase, others). However, membrane PD-L2 found on tumor cells was not associated with baseline peripheral blood markers (white blood cells, absolute neutrophil, lymphocyte, monocyte, and eosinophil counts, serum C-reactive protein, and serum Lactate Dehydrogenase [LDH], levels) in lung adenocarcinoma patients who underwent primary surgery. In a retrospective series of lung adenocarcinoma patients, including all the stages of the disease (from early to metastatic), PD-L2 was evaluated by IHC. Around 50% of the cases were stained PD-L2+ on tumor cells, with 30% resulting in PD-L2high (> 50% positive cells) and is associated with an EGFR mutation and a lower stage of the tumor. Longer progression-free survival was observed in patients with defined PD-L2+ (> 1% of positive cells). Different results were obtained in another more homogeneous retrospective cohort of patients (only those with resected lung adenocarcinoma were included), where the expression of PD-L2 by tumor cells (1% cut-off) was associated with worse outcomes (shorter disease-free survival and OS),^22^ probably related to the different patient population evaluated. In addition, in patients with resected squamous cell lung carcinoma, PD-L2 expression on tumor cells at two different cuts-off (> 5% and > 10% positive cells) predicted better outcomes. In renal cell carcinoma, PD-L2 on tumor cells (together with PCD-1 and PD-L1) was less expressed in primary tumors (16%) with respect to metastases (24%) even though this difference was not statistically significant. In histotypes with sarcomatoid transformation, amplifications of the Jak2/PD-L1/PD-L2 genes were detected. PD-L2 expression by tumor cells was further associated with a better prognosis in oropharyngeal squamous cell carcinoma and melanoma and with a worse prognosis in colorectal cancer, malignant salivary gland tumors, and chromophobe renal cell carcinoma. Different from PD-L1, cytoplasmic PD-L2 expression by IHC was found in 42% of patients with esophageal squamous cell carcinoma with no impact on disease-free survival. In breast cancer, PD-L2 expression by tumor cells was found in around 29% of the cases analyzed by IHC, with no clinically significant associations, findings that were conflicting with another study probably due to the different antibodies employed. Concerning the last subset of cells from the TME expressing PD-L2, the stromal cells, a contemporaneous overexpression of either PD-L2 or PD-L1 by Cancer-Associated Fibroblasts (CAFs) was observed as shown in human pancreatic carcinoma. These fibroblasts showed higher levels of PD-L1 and particularly of PD-L2 concerning the skin fibroblasts from healthy donors, playing an inhibitory role in the activity of Tumor-Infiltrating Lymphocytes (TILs).^23^ Remarkably, these CAFs had higher levels of PD-L2 regarding PD-L1, and blockade of the pathway through the use of anti–PD-L1 and anti–PD-L2 immune checkpoint blockade restored CD4+ (and with less frequency CD8+) T-cell proliferation. In mouse models, CAFs were shown to suppress T-lymphocyte function through the interaction of immune checkpoint pathways, like Fas/Fas Ligand (FasL) and PD-1/PD-L2. FASL and PD-L2 were more abundant in CAFs concerning normal fibroblasts, and when these ligands were blocked, the capacity of T-cells to exert their cytotoxic activity was rescued in an antigen-dependent manner. In lung tumors, there was an enrichment of FasL and PD-L2 in stromal areas, revealing the potential relevance of CAFs in the human TME and probably explaining some mechanisms of immune evasion that are mediated by stromal cells. PD-L1 and PD-L2 were constitutively expressed but also upregulated through IFN-γ in non–small cell lung cancer. Also, in head and neck cancer, PD-L1 and PD-L2 were observed in fibroblasts, with inhibitory actions on T lymphocytes. PD-L2 can also be expressed by mast cells, with immunosuppressive effects.^21^

## PD-1/PD-L1/PD-L2 as therapeutic targets and their significance in gynecological cancers

PCD-1 and its ligand PD-L1 are the best-described immune co-inhibitory molecules. The PD1 receptor can be expressed on CD8+ and CD4+ T-cells, whereas PD-L1 is expressed in activated T-cells, tumor-infiltrating macrophages or fibroblasts, and tumor cells.^24^ NK cells are important components of the innate immune system and play a central role in the TME design^25^ since they promote the adaptive response through their cytokine secretion polarizing T-cell activation.^26^ Moreover, the modulation of NK cell response can be controlled by the immune checkpoint molecules of the B7 family[8] and the most prominent checkpoint regulators are the abovementioned PCD-1/PD-L1, as well as CTL Antigen-4 (CTLA4). The role of B7-H6 in the inflammatory tumor microenvironment design was sought via the relationship of its expression with PD-L1 and NK-TILs status.^27^ The impressive results obtained with immunotherapy in other chemoresistant tumors such as melanoma and renal cancer have raised interest in the potential for using this approach in patients with gynecologic cancers. This has led to the development of a large number of clinical trials testing immunotherapy both as monotherapy and in combination with other strategies such as chemotherapy, targeted agents, or both. To date, only Microsatellite unstable (MSI)-H tumors and PD-L1-positive Cervical Cancer (CC) have received Food and Drug Association (FDA) approval for treatment with immune checkpoint inhibitors. PD-L1 is important in terms of potential immunotherapy in tumors, including gynecological tumors. On one hand, gynecological tumors are important because of their high incidence and mortality worldwide. CC is the fourth most commonly diagnosed tumor and a fourth most common cause of death but is on high second place in the incidence and mortality among women in low-income countries. Ovarian Cancer (OC) is represented by an incidence of 1.6% among all cancers, overall mortality of 1.3%. Endometrial Cancer (EC) is present with an incidence of 2.1% among all malignancies, and as a cause of death in 0.9 % of all cancer deaths.^28^ It is already recognized that gynecological cancers do not respond very well to classic chemotherapy. The only exception is perhaps OC which can be platinum-sensitive. That's why the new therapeutical approach including immunotherapy and recognition of immunomodulating checkpoints like PCD-1/PD-L1 is extremely important. Immunotherapy has already shown results in some malignant tumors like melanoma and renal cancer which are also chemoresistant, and that raised interest in using this type of therapy in gynecological cancers. The expression of PCD-1/PDL-1 in these tumors is the exact reason for using this therapy approach and the rationale for immunotherapy in different gynecological malignancies.^29^

Ovarian carcinomas include five major and distinct histological types with different characteristics and prognoses: high-grade serous carcinoma (HGSC, 70%), low-grade serous carcinoma (LGSC, < 5%), endometrioid carcinoma (EC, 10%), clear-cell carcinoma (CCC, 10%) and mucinous ovarian carcinoma (MOC, 3%). 1 HGSC and CCC are of poorer prognoses. The Cancer Genome Atlas (TCGA) project identified genetic abnormalities or susceptibility alleles for the most common OCs and suggested several subtypes, including an immunoreactive subtype characterized by expression of the T-cell chemokine ligands more specifically identified in HGSC. Several studies have focused on inflammatory infiltrate, T-cells, and TAM expression on both OC cell lines and in vivo. PCD1 is implicated in programmed cell death and PCD1/ PD-L1 is an important immune checkpoint in the proliferation and development of tumors. Tumor cells with PD-L1 transmembrane protein bind to the PD-1 receptor of T-lymphocytes and inactivate them. Treatments that block the PCD-1 receptor or the PD-L1 protein (anti-PCD-1 or anti-PD-L1) can reverse the inactivation of T-lymphocytes. These immune cells can subsequently have a tumor cell action. It has recently been suggested that the presence of intratumoral inflammatory infiltration associated with PD-L1 expression influences survival in HGSC with clinical trials using anti-PD-L1 immunotherapy such as pembrolizumab and avelumab showing promising results. Two recent meta-analyses suggested that PD-L1 expression was not linked to tumor histology, OS, and Progression-Free Survival (PFS), but that PD-L1 mRNA expression was closely correlated with poor PFS. However, immunohistochemistry evaluation of PD-L1 before treatment was not always performed in these studies. Furthermore, in contrast to non-small-cell lung cancer, there is currently a lack of a consensual interpretation score for PD-L1 in OCs. Moreover, the published threshold of positivity is variable^18^ giving rise to an extensive debate about the prognostic and predictive values of response to treatment of PD-L1 expression. To date, there are no recommendations for evaluating immunohistochemical PD-L1 expression for targeted therapy in the first-line treatment of OCs (INCa).^30^ But some studies confirm the rationale for using immunotherapy in ovarian cancer patients. PD-1/PD-L1 expression in OC is the main rationale for using immunotherapy in this malignancy. Also, it is observed that higher expression of these immune checkpoints is associated with poor prognosis and survival. Some investigators showed that association between PD-L1 expression and peritoneal dissemination, suggesting that PD-L1 could be the most important target for immunotherapy. It is believed that PD-L1 expression represents an anti-tumor response in the body and its correlation with the clinical outcome has been proven.^30^

The rationale for immunotherapy and its correlation with PD-L1 expression is proven in endometrial carcinoma as well. It is shown that endometrium specifically correlates with the immune system and that the role of immunity in endometrial tissue is protection from sexually transmitted diseases. On the other hand, immunity may be a target for immunotherapy, especially because EC represents histology that often expresses new checkpoints. However, PD-L1 expression is found in tumor cells in 61% of EC cases and even 80% of the cells. The same authors confirmed in their work that metastatic EC expressed PD-L1 in tumor cells in 100% of cases.^30^

When it is to CC CTLA4 and PD-L1 expression is found in dendritic cells in Cervical Intraepithelial Neoplasia (CIN) samples.^31^ PCD1/PDL1 expression is found in even 95% of epithelial lesions and approximately 80% of squamous carcinomas.^31^ Over 70% of cervical cancer cases diagnosed in developing countries are locally invasive or metastatic, contributing to the high mortality rate of cervical cancer. The 5-year OS rate of local cervical cancer can achieve approximately 75%–85% through effective treatment modalities such as surgery, CCRT, etc. Nevertheless, the 5-year OS of recurrent, persistent, metastatic cervical cancer is only approximately 15%. The poor prognosis is mainly due to limited therapeutic options. The majority of these patients can only be treated with palliative chemotherapy, in which platinum-based chemotherapies were the prior choice. In 2014, the GOG 240 trial indicated that when bevacizumab was added to the chemotherapy, the ORR was improved from 36% to 48%, and the OS could be prolonged from 13 to 17 months for recurrent, persistent, metastatic cervical cancer, thus laying the foundation for the first-line choice of combining bevacizumab with chemotherapy for this population. However, for those who progress during the first-line treatment, the lack of effective second-line treatment remains to be the main reason for the high mortality rate. Currently, immune checkpoint inhibitors, especially PCD-1/PD-L1 inhibitors, have achieved favorable efficacy in treating multiple solid tumors, including cervical cancer. Numerous immunomodulatory therapies are being investigated in clinical trials with diverse potential targets, including PCD- 1/PD-L1, CTLA-4, Tim-3, ICOS, 4-1BB, and OX-40. Among these novel targets, ICOS, 4-1BB, and OX-40 are costimulatory receptors, while PD-1/PD-L1, CTLA-4, and Tim-3 are negative immune regulators of T-cells. Currently, only CTLA-4 inhibitors and PD-1/PD-L1 inhibitors have been approved by the FDA. CTLA-4 integrates with the costimulatory molecules CD80 (B7-1) and CD86 (B7-2) that express on the surfaces of APCs, while PD-L1 is expressed on a wide variety of cell types, including tumor-associated fibroblasts, tumor cells, APCs, etc. As a result, CTLA-4 inhibits T-cell activation within secondary lymphoid organs, but PCD-1/PD-L1 chiefly regulates T-cell function within peripheral tissues and the tumor microenvironment. Therefore, PCD-1/PDL1 signaling is more specific to the tumor than CTLA-4 signaling, and inhibitors of PCD-1/PD-L1 may cause less damage to healthy tissue. To date, numerous studies have investigated the expression of PD-L1 in CC. The expression of PD-L1 has been reported in 34.4%–96.0% of CC tissues, while expression of PD-L1 in histologically normal cervical tissues was rarely found. It is showed that PD-L1 expression was positive in 32 of 93 (34.4%) CC samples, subcategorically in 28 of 74 (37.8%) SCCs, 2 of 7 (28.6%) adenosquamous carcinomas, and 2 of 12 (16.7%) endocervical adenocarcinomas. Also, PD-L1 expression was found in 96% of the samples. Specifically, for cervical SCC, PD-L1 expression was found in 80% (56/70) of cases. In the TCGA database for cervical SCCs, the amplification or gain of PD-L1 was found in 28 of 129 (22%) cases. In addition, PD-L1 can also be expressed on TILs, which plays a role in antitumor response inhibition. A study found that for cervical SCCs samples, the expression rates of PD-L1 on cancer cells and TILs were 59.1 and 47.0%, respectively. Collectively, these data suggest that both PD-L1 and PD-1 are widely expressed in cervical cancer tumor cells and stroma, providing potential therapeutic targets for PD-1/PD-L1 inhibitors. Notoriously, persistent HPV infection is involved in the pathogenesis of cervical cancer and is related to its prognosis. Several teams have interrogated whether HPV infection could affect PD-L1 expression in cervical cancer and found that HPV positivity was positively correlated with increased PD-L1 expression. Considerable effort has been made to dissect the underlying mechanism of the association between HPV status and PD-L1 expression in HPV-related solid tumors, mainly HNSCC and cervical cancer. In HPV-HNSCCs, membranous expression of PD-L1 and significantly increased levels of mRNA of IFN-γ were found in the tonsillar crypts, As tonsillar crypts witnessed the initial HPV infection, and IFN-γ induces PD-L1 expression, this evidence might support the role of the PCD-1/PD-L1 interaction in creating an “immune-privileged” site for initial viral infection and subsequent adaptive immune resistance.^31^ Therefore, regardless of the pathogenesis of this tumor which is associated with papillomavirus infection in the majority of cases, immunology, PCD1/PD-L1 expression, and immunotherapy play a very important role in future understanding and management of the disease.^29^

## PD-1/PD-L1/PD-L2 and their role in other tumors

Checkpoint blockades are registered for the treatment of various cancers, such as advanced clear-cell renal carcinoma with intermediate and poor prognosis, advanced metastatic non-small cell lung carcinoma, recurrent Hodgkin's lymphoma, and recurrent hepatocellular carcinoma, following the failure of other treatments.^32^ PCD1/PD-L1 drugs are approved by FDA for the therapy of lung cancer (Nivolumab, Pembrolizumab, Atezolizumab, and Durvalumab), and their efficacy in treatment is shown in different clinical trials.^33^ Also, studies have shown that PCD1/PD-L1 pathway antagonists can induce sustained clinical responses in some cases with metastatic negative breast cancer. Studies also tend to show if immunotherapy would be beneficial here as adjuvant and neoadjuvant treatment.^33^

PCD-1/PD-L1 is a transmembrane immunoglobulin-like protein that is expressed in the thymus and spleen. It is hard to detect in peripheral leucocytes, except in activated T and B cells. Its role is negative regulation of immune response – shown for the first time in experimental mice. The absence of PCD-1 expression is shown to have caused a higher incidence of autoimmune diseases, such as lupus-like proliferative glomerulonephritis, arthritis, and dilated cardiomyopathy. Its ligand, PD-L1, was discovered in later years. Administration of this ligand to activated T-cells resulted in the inhibition of their proliferation and the reduction of secretion of IFN-γ and interleukin. In physiological conditions, PD-L1 is expressed in T- and B-cells, dendritic cells, macrophages, epithelial cells, myocardial cells, pancreatic islands cells, keratinocytes, and the maternal-fetal barrier. IFN-γ increases its expression.^33^

The expression of PD-L1 on normal tissue is limited. However, numerous tumor cells show overexpression of PD-L1, and as it is mentioned above, it can be a target for immunotherapy.^7^ In a study including 42 cases with different malignancies who had received PD-L1 antibodies, PD-L1 positive tumors exhibited a significantly better response to the therapy than PD-L1 negative tumors. A subsequent study showed that the expression of PD-L1 in more than 50% of tumor cells was associated with a better response to Pembrolizumab therapy in patients with advanced non-small cell lung cancer. In other studies that included patients with renal cell carcinoma or recurrent head and neck cancer treated with nivolumab, it was shown that expression of PD-L1 was a significant predictor of nivolumab efficacy.^34^

The interaction of PCD-1 expressed on T-cells with PD-L1 on the membrane of cancer cells leads to T-cell exhaustion and inhibits the subsequent immune reaction, bypassing immune surveillance. Besides, it has been found that PD-L2 is another ligand that interacts with PCD-1, further suppressing T-cell proliferation and cytokine release. Until now, the specific mechanism of PD-L1 and PD-L2 regulation in tumor immune escape remains largely elusive. As is shown, the effects and mechanism of antibodies to PD-1 and its ligands have been investigated in numerous malignancies, including hepatocellular carcinoma. It has been reported that CD8-positive T-cells promoted PD-L1 expression on HCC cells in IFN-γ dependent manner, leading to T-cell apoptosis. PD-1 expression was significantly elevated in CD8+ and CD4+ cells obtained from HCC tissue compared to control tissue. Antibodies to PD-L1, TIM3, and LAG3 elicit reactions of HCC-derived T-cells against tumor antigens, which might become an essential strategy for HCC treatment, too. In addition, PCD-1 expression in HCC has also been suggested to increase tumor proliferation independently of adaptive immunity, interacting with a downstream target of rapamycin effectors eukaryotic initiation factor 4E and ribosomal protein S6. Moreover, PCD-1 checkpoint blockade in combination with rapamycin inhibitor results in more durable and synergetic tumor regression. In terms of prognosis, just like it is mentioned above, high expression of PD-L1 and PD-L2 indicated poor prognosis.^35^

Multiple studies have reported that immune cell infiltration was related to a tumor in many aspects. Considering the matrix of gene expression, it generates CD3+ T-cells, B lymphocytes, cytotoxic lymphocytes, NK cells, CD8+ cells, cells derived from monocytes, myeloid dendritic cells, neutrophils, endothelial cells, and fibroblasts for each sample. Some studies showed that PCD-1 and PD-L2 expression had been associated with the recurrence of HCC. However, none of the three genes showed a significant correlation with the clinical stage.^35^ Other studies demonstrated significant relation between PD-L1 expression and multiple markers of cancer aggressiveness including satellite nodes, macrovascular invasion, microvascular invasion, and poor differentiation. Besides, PCD-1 and PD-L1 expression was upregulated in HCC tissues compared with normal tissue, which was positively related to clinical stage and lymph node metastasis, but negatively related to the survival of HCC patients. These results suggest that the PCD-1/PD-L1/PD-L2 axis might correlate with multiple clinical parameters of HCC. The same authors investigating genes correlation with the above-mentioned axis showed that PD-L1/PD-L2 interacted with 7 genes while PD-L1 showed co-expression with 39 genes. PCD-1-related genes were mainly enriched in T-cell activation, lymphocytes activation, leukocyte cell-cell adhesion, cytosolic calcium ion concentration, T-cell signaling pathway, calcium ion homeostasis, the release of sequestered calcium ion into the cytosol, and cellular defense response, cell activation, cell-cell adhesion, IFN-γ -mediated signaling pathway, negative regulation of activated T-cell proliferation, interleukin 10 production, and response to hyperoxia. As indicated by the enrichment analysis, PCD-1/PD-L1/PD-L2 signaling is a key regulator of T-cell activation and other important lymphocyte functions. The same study reported that CD8+ cells greatly increase PD-L1 expression on cancer cell lines, and PD-L1 expression and CD8+ T-cell density showed a significant positive correlation in HCC patients.^35^, ^36^, ^37^, ^38^, ^39^

## Conclusion

Recently, cancer immunotherapy targeting PCD-1 or PD-L1 has proven effective in causing durable antitumor immune responses with less toxicity in many types of tumors, the authors believe that PCD-1/PD-L1 blockade therapy will be the major cancer immunotherapy method in the next few years, even though there is still much to be learned about this signaling pathway. Key questions remaining to be resolved include: i) How to select PD-1/PD-L1-positive patient groups? ii) What are the features of these patients, and what efficient clinical detection method should be used? iii) How can the abundance of tumor-infiltrating CD8+T-cells be increased in TME, particularly CD8+T-cell that display tumor-reactive intratumoral TCR repertoires, but not bystander CD8+TILs that can not kill the tumor cell? iv) What is the mechanism by which PD-1 regulates on CTLs and Tregs, and what is the mechanism by which PD-L1 acts on tumor cells and APC cells in TME, and can we find more efficient inhibitors based on the mechanism? v) Do we possess any good treatment for PD-1/PD-L1- negative patients? vi) Can other therapies or combination therapies with an anti-PD antibody approach be optimal for these patients? Consequently, with a deeper understanding of personal genomic information, personalized markers in guiding anti-PD therapy alone or with other targets will be critical to achieving clinical results of such therapies, and more work needs to be performed in order to achieve clarity regarding these key questions. Similar to the tip of an iceberg, PCD-1/PD-L1 blockade antitumor immunotherapy opens a new era of cancer treatment, and further work on safety and efficiency will be required.

## Data availability

The clinical data used to support the findings of this study are available from the corresponding author upon request.

## Author's contribution

Bojana Gutic: Conceptualization, data curation, formal analysis, investigation, methodology, project administration, resources, validation, visualization, writing-original draft.

Tatjana Bozanovic: Investigation, methodology, project administration, resources, validation, visualization.

Aljosa Mandic: Investigation, methodology, project administration, resources, validation, visualization.

Stefan Dugalic: Investigation, methodology, project administration, resources, validation, visualization.

Jovana Todorovic: Investigation, Methodology, Project administration, Resources, Validation, Visualization.

Dejana Stanisavljevic: Investigation, Methodology, Project administration, Resources, Validation, Visualization.

Miroslava Gojnic Dugalic: Investigation, Methodology, Project administration, Resources, Validation, Visualization.

Demet Sengul: Investigation, Methodology, Resources, Software, Supervision, Validation, Visualization, Writing-review&editing.

Dzenana A. Detanac: Investigation, Methodology, Validation, Visualization, Writing-review&editing.

Ilker Sengul: Investigation, Methodology, Project administration, Resources, Software, Supervision, Validation, Visualization, Writing-review&editing.

Dzemail Detanac: Investigation, Methodology, Validation, Visualization, Writing-review&editing.

José Maria Soares Junior: Investigation, Methodology, Validation, Visualization, Writing-review&editing.

## Funding statement

None declared.

## Conflicts of interest

The authors declare no conflicts of interest.
